# What is the mid-wall linear high intensity “lesion” on cardiovascular magnetic resonance late gadolinium enhancement?

**DOI:** 10.1186/s12968-020-00665-5

**Published:** 2020-09-14

**Authors:** Masashi Nakamura, Tomoyuki Kido, Kuniaki Hirai, Kohei Tabo, Yuki Tanabe, Naoto Kawaguchi, Akira Kurata, Teruhito Kido, Osamu Yamaguchi, Teruhito Mochizuki

**Affiliations:** 1 Department of Radiology, Ehime University Graduate School of Medicine, Shitsukawa, Toon, Ehime 791-0295 Japan; 2 Department of Cardiology, Ehime University Graduate School of Medicine, Shitsukawa, Toon, Ehime 791-0295 Japan

**Keywords:** Anterior septal perforator artery, Contrast enhancement, Linear high intensity, Late gadolinium enhancement, Cardiovascular magnetic resonance

## Abstract

**Background:**

Cardiovascular magnetic resonance (CMR) late gadolinium enhancement (LGE) is a valuable technique for detecting myocardial disorders and fibrosis. However, we sometimes observe a linear, mid-wall high intensity signal in the basal septum in the short axis view, which often presents diagnostic difficulties in the clinical setting. The purpose of this study was to compare the linear, mid-wall high intensity in the basal septum identified by LGE with the anterior septal perforator arteries identified by coronary computed tomography angiography (CorCTA).

**Methods:**

We retrospectively selected 148 patients who underwent both CorCTA and CMR LGE within 1 year. In the interpretation of LGE, we defined a positive linear high intensity (LHI+) as follows: ① LHI in the basal septum and ② observable for 1.5 cm or more. All other patients were defined as a negative LHI (LHI-). In LHI+ patients, we assessed the correlation between the LHI length and the septal perforator artery length on CorCTA. We also compared the length of the septal perforator artery on CorCTA between LHI+ patients and LHI- patients.

**Results:**

A population of 111 patients were used for further analysis. Among these , there were 55 LHI+ patients and 56 LHI- patients. In LHI+ patients, linear regression analysis revealed that there was a good agreement between LGE LHI and septal perforator arteries by CorCTA in terms of length measurements. The measured length of the anterior septal perforator arteries was significantly shorter in LHI- patients than in LHI+ patients (10 ± 8 mm vs. 21 ± 8 mm; *P* < 0.05).

**Conclusions:**

The LHI observed in the basal septum on short axis LGE may reflect contrast enhancement of the anterior septal perforator arteries. It is important to interpret this septal LHI against knowledge of anatomic structure, to avoid misinterpretations of LGE and prevent misdiagnosis.

## Introduction

Cardiovascular magnetic resonance imaging (CMR) is a very useful tool that can noninvasively evaluate cardiac anatomy, function, and blood flow [1, 2]. Myocardial viability is commonly assessed using late gadolinium enhancement (LGE). CMR LGE is a valuable technique that makes it possible to diagnose myocardial fibrosis and disorders noninvasively [3–5]. The most widely used pulse sequence type of LGE is inversion recovery (IR) with magnitude reconstruction, which usually obtains an image 10–15 min after administration of gadolinium contrast agent [6, 7]. About 10 min after injection of the contrast medium, this medium is evenly distributed in extracellular or intravascular space and is in equilibrium. With LGE, infarcted myocardium or fibrosis show relatively high signal intensity as compared with normal myocardium, due to increased stroma. Therefore, this technique is used as a powerful option for the evaluation of non-ischemic cardiomyopathy, because it allows the differential diagnosis of cardiomyopathy, with high probability, based on various patterns of LGE [8–13].

However, CMR produces some artifacts due to the direct effect of myocardial wall motion, or the indirect effect of shortening acquisition time, to eliminate movement [14]. In LGE studies, choosing the correct inversion time (TI) to null normal myocardium can be challenging for inexperienced operators. If TI is not adequate, the normal myocardium will not be nulled, the contrast between infarcted and normal myocardium will be reduced, and additional misleading artifacts will be produced. Due to these effects, we often observe some high intensity signals on LGE imaging, which are believed to be artifacts.

**Fig. 1:**
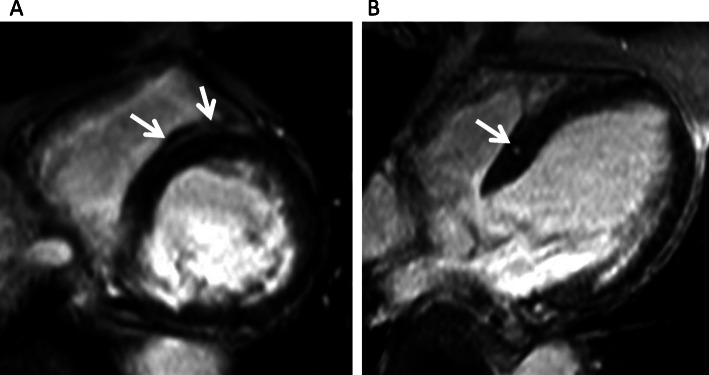
A mid-wall, linear, high intensity (LHI) in the basal septum in the short axis view (**a**) and a round-shape high intensity in the orthogonal long axis view (**b**)

In particular, we sometimes experience a linear, mid-wall high intensity in the basal septum in the short axis view, which often poses diagnostic difficulty in the clinical setting. Such linear high intensity (LHI) appears as round-shaped high intensity in the orthogonal long axis view and is thereby clearly distinguishable from right ventricular (RV) blood pools behind RV trabeculation [15] or artifacts (Fig. 1). The linear mid-wall LGE in the septum is also a pattern of LGE that is found in dilated cardiomyopathy (DCM) [16]. In addition, mid-wall fibrosis in DCM, as determined by LGE, is a predictor of the combined end point of all-cause mortality and cardiovascular hospitalization, which is independent of ventricular remodeling [17]. Therefore, it is clinically very important to distinguish the LHI found in the septum from mid-wall fibrosis in patients with DCM. However, no previous papers have discussed this issue.

In recent years, multidetector computed tomography (CT) with submillimeter collimation and high-speed gantry rotation has allowed the representation of thinner vessels, such as septal branches, with high temporal resolution and isotropic voxels [18].

**Fig. 2:**
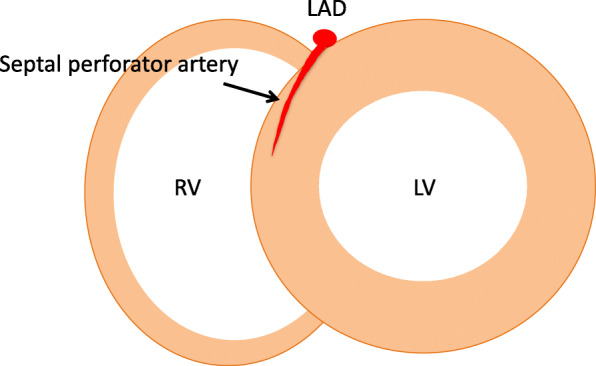
A representative image of the anterior septal perforator artery in the short axis view. LV, left ventricle; RV, right ventricle; LAD, left anterior descending coronary artery

We noticed that the LHI observed in the short axis view on CMR LGE was anatomically close to the course of the anterior septal perforator arteries on coronary CT angiography (CorCTA) (Fig. 2). The anterior septal perforator arteries originate from the proximal part of the left anterior descending coronary artery (LAD) and irrigate two-thirds of the upper part of the interventricular septum. They vary in number, with an average of eight branches; the first septal artery is usually the largest and the longest [19, 20].

A recent case report demonstrated that the basal septal perforator vessel can mimic late iodine enhancement in delayed-phase cardiovascular CT [21]. On the other hand, since LGE imaging is performed when the signal of normal myocardium becomes null, it is considered that a blood vessel lumen running in the myocardium is rendered with a relatively high signal. We therefore hypothesized that the mid-wall LHI on LGE may represent contrast enhancement of the anterior septal perforator arteries. To investigate this hypothesis, we compared the linear, mid-wall LHI lesions identified on LGE in the basal septum with the anterior septal perforator arteries identified on CorCTAto determine the relationship between them.

## Material and methods

### Study populations

The study was reviewed and approved by the institutional review board of our university. This study was designed as a retrospective chart review; as such,written patient consent was waived.

From the clinical database, we retrospectively selected 433 consecutive patients who underwent CMR between November 2013 and September 2017. In that group, we selected 148 patients who underwent both CorCTA and CMR LGE within 1 year (median, 28 days; first quartile–third quartile, 13–71 days). The exclusion criteria were as follows: (1) obvious LGE in the basal septal due to primary disease, (2) poor LGE image quality or CorCTA. Of 148 patients, 37 patients were excluded according to the set criteria. The details of these excluded patients were as follows: 31 patients showed LGE in the septal region (15 patients due to old myocardial infarctions, nine patients due to hypertrophic cardiomyopathy, three patients with DCM, two patients with amyloidosis, one patient with sarcoidosis, one patient with hypertensive heart), three patients had poor CMR image quality due to poor breath holding or high heart rate, and three patients had poor CorCTA image quality due to motion artifacts resulting from high heart rate.

Finally, 111 patients were used for further analysis (mean age, 61 ± 15 years, men, 65%, Table 1).

**Table 1:** Clinical characteristics of the patient population

Characteristics	LHI+	LHI-	*P* value
The number of patients	55	56	
Mean age (years)	61 ± 12	62 ± 17	0.15
Male sex	41 (75%)	31 (55%)	0.03
Body mass index (kg/m^2^)	24 ± 3	23 ± 4	0.10
Cardiovascular risk factors			
Diabetes	15 (27%)	12 (21%)	0.47
Smoking	31 (56%)	24 (43%)	0.15
Dyslipidemia	26 (47%)	26 (46%)	0.92
Hypertension	34 (62%)	27 (48%)	0.15
Family history of CAD	18 (33%)	22 (39%)	0.47
Baseline heart rate (beats/min)	65 ± 10	66 ± 9	0.66
LVEF (%)	56 ± 12	55 ± 10	0.28
LVEDV (mL)	124 ± 38	125 ± 42	0.15
LVESV (mL)	56 ± 41	56 ± 41	0.58
Clinical diagnosis			
Ischemic heart disease	38 (69%)	39 (69%)	0.93
Cardiomyopathy	6 (11%)	7 (13%)	0.55
Other	11 (20%)	10 (18%)	0.67

Data are presented as mean ± standard deviation or number (%) of subjects

*LHI* linear high intensity, *LVEF* left ventricular ejection fraction, *LVEDV* left ventricular end-diastolic volume, *LVESV* left ventricular end-systolic volume, *CAD* coronary artery disease

### Data acquisition

#### CMR

All studies were performed on a clinical 3 T CMR scanner (Achieva Quasar Dual; Philips Healthcare, Best, The Netherlands) equipped with a dedicated cardiac software package and a 32-element cardiac phased-array coil (16 posterior elements, 16 anterior elements); a 4-lead vector electrocardiogram (ECG) was used for cardiac gating. In all patients, double-angulated scout images were obtained to plan cardiac axis views, and retrospective ECG gated cine imaging was performed using a segmented balanced steady state free precession (bSSFP) sequence in continuous short-axis views, spanning the entire left ventricle (LV) from base to apex. Ten minutes after the injection of 0.2 mmol/kg gadopentetate dimeglumine (Magnevist; Bayer Healthcare, Berline, Germany), we acquired three-dimensional (3D) IR sequences that spanned the LV from the base to the apex, selecting the myocardium null TI values from the TI scout images. The imaging parameters were: repetition time/echo time, 3.4/1.6 ms; TI, 300–400 ms; flip angle, 15°; field-of-view, 350 mm × 350 mm; pixel size, 1.6 mm × 2.3 mm; slice thickness, 10.0 mm; sensitivity encoding (SENSE) factor, 2.4.

#### Coronary computed tomography angiography

We used a 256-slice (128 multi-detector row) CT scanner (Brilliance iCT; Philips Healthcare, Cleveland, Ohio, USA). All patients received 0.6 mg of nitroglycerin (Myocor; Astellas Pharma, Tokyo, Japan), and intravenous beta-blocker (Corebeta; landiolol hydrochloride, 0.125 mg/kg, Ono Pharmaceutical Co., Ltd., Tokyo, Japan), 5 min prior to the timing bolus scan, to reduce heart rate if the heart rate was > 75 beats per minutes, in the absence of contraindication. CorCTA was performed with iohexol (Omnipaque; 350 mg iodine/mL; Daiichi Sankyo, Tokyo, Japan) or with iopamidol (Iopamiron; 370 mg iodine/mL; Bayer Yakuhin, Osaka, Japan) at an injection rate of 5.0–5.5 mL/s for 10 s, followed by a saline flush (20 mL, 5.0–5.5 mL/s). The scan parameters were as follows: retrospective ECG-gated mode (heart rate > 60 beats/min) or prospective ECG-gated mode (heart rate ≤ 60 beats/min); tube voltage, 120 kV; effective tube current time-product, 800–1300 mAs/rotation with dose modulation; gantry rotation time, 0.27 s/rotation; collimation, 2 × 128 × 0.625 mm with a dynamic z-focal spot; 250-mm display field-of-view; 0.8/0.4-mm slice thickness/overlap; and 512 × 512 image matrix.

### Image analysis

#### Late gadolinium enhancement

All short-axial LGE images were evaluated by a cardiovascular radiologist with 12 years of CMR experience. In the interpretation of LGE, we defined a positive LHI (LHI+) as follows: a ① LHI in the basal septum, ② observable at 1.5 cm or more. In this study, 1.5 cm was set as the cut off length that can be definitely claimed not to be an artifact visually. All other patients were defined as a negative LHI (LHI-). In all patients, two radiologists decided by consultation whether LHI was present or not. For LHI+ patients, the LHI length was measured.

As an initial pilot study, we compared 21 patients with mid-wall fibrosis who were clinically diagnosed with DCM in our hospital as well as patients with LHI+ patients.

#### Coronary computed tomography angiography

We divided the LV myocardium into 16 equal parts from the base to the apex in the longitudinal direction, and created short-axis maximum intensity projection (MIP) CT images of the same slice as in CMR LGE. For all patients, another cardiovascular radiologist with 3 years of CorCTA experience decided on the presence or absence of an anterior septal perforator artery on CorCTA and measured its length.

We used the dedicated SYNAPSE VINCENT software package (Fujifilm Corp., Ltd., Tokyo, Japan) for measuring LHI length and perforator arteries.

### Statistical analysis

Continuous data are shown as the mean and standard deviation (SD) or as the median (first quartile, third quartile). Independent Student’s t tests were used to compare differences in continuous data. Differences in proportions were assessed using the chi-square test or Fisher’s exact test. In LHI+ patients, we assessed the correlation between LHI length on CMR LGE and that of the septal perforator artery on CorCTA. We also compared the length of the septal perforator artery on CorCTA between LHI+ and LHI- patients. Spearman correlation coefficients were used to assess the correlation between the lengths of the LHI and the anterior septal perforator artery. Wilcoxon matched-pairs signed-rank tests were used to compare the length of the anterior septal perforator arteries on CorCTA between LHI+ and LHI- patients. *P* < 0.05 was considered statistically significant. All statistical analyses were performed using commercially available software (JMP version 12; SAS Institute, Cary, North Carolina, USA).

## Results

**Fig. 3:**
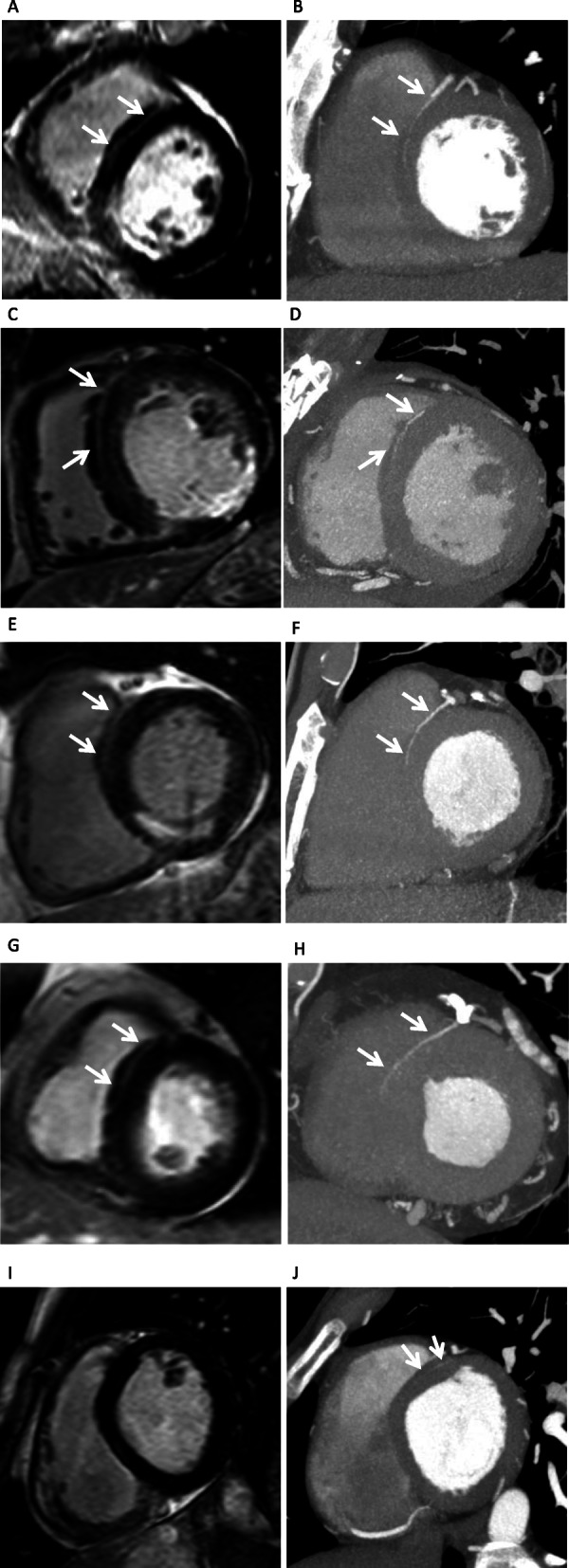
**a-j**. Some patients of linear high intensity (LHI) in late gadolinium enhancement (LGE) cardiovascular magnetic resonance (CMR) and anterior septal perforator arteries in coronary computed tomography angiography (CorCTA). **a-b**. Fifty-six-year-old, male, acute inferior myocardial infarction. **a** LHI on CMR LGE, **b** anterior septal perforator arteries on CorCTA. C-D. Forty-eight years old, male, old inferior myocardial infarction. **c** LHI on CMR LGE, **d** anterior septal perforator arteries on CorCTA. E-F. Fifty-eight-year-old, male, old inferior myocardial infarction. **e** LHI on CMR LGE, **f** anterior septal perforator arteries on CorCTA. G-H. Seventy-one-year-old, female, acute anterior myocardial infarction. **g** LHI on CMR LGE, **h** anterior septal perforator arteries on CorCTA. **i-j**. LHI could not be identified (negative LHI) on LGE **i**. However, CorCTA could detect very thin anterior septal perforator arteries with higher spatial resolution **j**

**Fig. 4:**
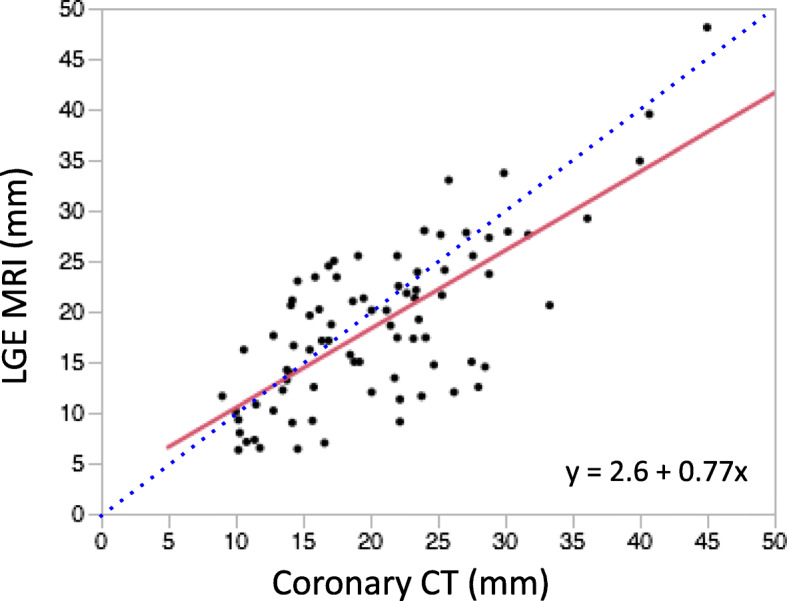
Linear regression showed a good correlation between LHI length measurements on CMR LGE and septal perforator arteries on CorCTA

**Fig. 5:**
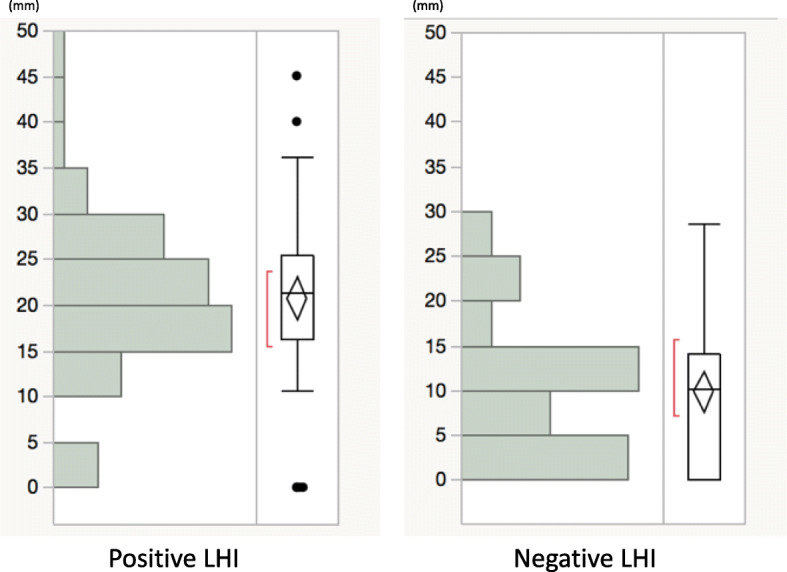
The measured length of anterior septal perforator arteries by CorCTA between LHI+ patients (**a**) and LHI- patients (**b**)

Among the 111 patients, there were 55 LHI+ and 56 LHI- patients. We observed that the LHI on LGE and anterior septal perforator arteries on CorCTA were very similar in shape and running direction (Fig. 3a-h). The measured length of the anterior septal perforator arteries on CorCTA was significantly longer than that in LHI on CMR LGE (16 ± 10 mm vs. 14 ± 10 mm; *P* < 0.05) (Fig. 3i, j). A linear regression analysis revealed that there was good agreement between LHI length on LGE and that of septal perforator arteries on CorCTA (R^2^ = 0.53, *P* = 0.13; Fig. 4). Among the 55 LHI+ patients, anterior septal perforator arteries were seen in the same region on CorCTA in 53 patients (96%) (Table 2). On the other hand, in the 56 LHI- patients, anterior septal perforator arteries were identified in only 39 patients on CorCTA (70%). The measured length of the anterior septal perforator arteries was significantly shorter in LHI- patients (10 ± 8 mm vs. 21 ± 8 mm for LHI+; *P* < 0.05) (Fig. 5).

**Table 2:** Patient number of cardiovascular magnetic resonance (CMR) late gadolinium enhancement (LGE) and coronary computed tomography angiography (CorCTA)

	CorCTA+	CorCTA-	Total
CMR (LHI+)	53	2	55
CMR (LHI-)	39	17	56
Total	92	19	111

*LHI* linear high intensity, *CorCTA+* patient in which an anterior septal perforator artery could identified on coronary CT, *CorCTA-* patient in which an anterior septal perforator artery could not be identified on coronary CT

**Fig. 6:**
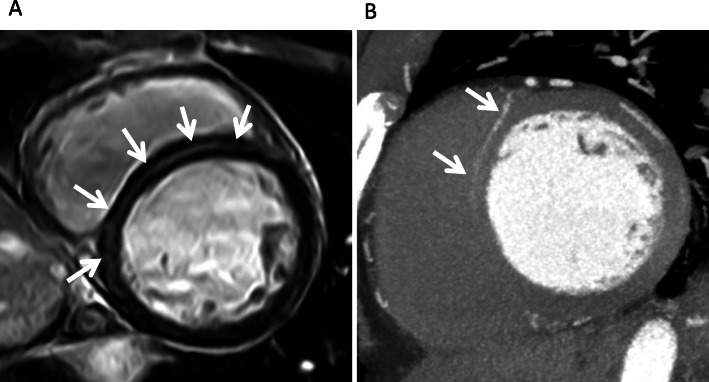
Forty-six-year-old, male, dilated cardiomyopathy. Typical mid-wall fibrosis is seen in the mid-layer of the basal septum from the anterior to the inferior side (**a**; arrow). The LGE pattern is distinctly different from that of the anterior septal perforator artery observed using CorCTA on another day (**b**; arrow)

Compared with mid-wall fibrosis in DCM, LHI has the following characteristics: 1) It runs on the epicardial side of the septum (one-third on the adventitial side of the septum) in 98% (54/55) of the patients with LHI and in 10% (2/21) of the those with DCM-related mid-wall fibrosis; 2) It starts from the anterior interventricular sulcus in 96% (53/55) of patients with LHI and in 24% (5/21) of those with DCM-related mid-wall fibrosis; 3) It stays in the anterior septum (does not reach the inferior septum) in 91% (50/55) of the patients with LHI and in 19% (4/21) of those with mid-wall fibrosis of DCM (Fig. 6).

## Discussion

CMR LGE is a useful tool for scar detection, based on the even distribution of contrast medium in the extracellular or intravascular space. However, with the increasingly high resolution of CMR in recent years, a high intensity, which has been considered to be nonspecific, has also been frequently observed [14]. It has not previously been described what this septal LHI seen on LGE represents. Our report demonstrated that the LHI found in the basal septum is likely to represent a normal septal perforator artery.

In the present study, anterior septal perforator arteries were identified at a high rate by CorCTA among LHI+ patients, and the length of these features correlated well. Also, their morphology and running directions were very similar. On the other hand, for LHI- patients, the anterior septal branch length measured on CorCTA was significantly shorter. Based on these results, we considered that the LHI observed in the basal septum in short-axis LGE images reflects contrast enhancement of the anterior septal perforator arteries.

In this study, LHI by CMR was visually confirmed as anterior septal perforator arteries on CorCTA in LHI+ patients. Since all LGE images were obtained using a 3D sequence, the septal high intensity could be identified well due to the high spatial resolution, high signal-to-noise ratio, and gapless slices. On the other hand, the measured length of the anterior septal perforator arteries on CorCTA was significantly longer than the LHI on CMR LGE. The reason may be that CorCTA has a higher spatial resolution than CMR, as shown in Fig. 3 (i) and (j), and could measure vessels to the periphery. In 56 of 111 patients, anterior septal perforator arteries were found on CorCTA, but these patients were defined as LHI- by CMR. Given that the mean length of the anterior septal perforator arteries on CorCTA was 10 ± 8 mm in LHI- patients, these arteries may not have reached the set LHI criteria (observable for 1.5 cm or more) in this study. It is possible that the LHI described here may be visualized increasingly with improvement in the spatial resolution and signal-to-noise ratio of CMR in future. Although in a small number (2 of 55 patients), for some patients that were defined as positive LHI by CMR, no marked anterior septal perforator arteries could be identified by CT. The LV myocardium includes not only the septal artery, but also vein, capillaries, arterio- and venoluminal vessels, and sinusoids, etc. [22]. Although very rare, these structures might also be recognized as LHI by LGE. However, we believe that the septal LHI is likely to be an artery, and not a vein, for the following reasons: first, it is very similar to the running of the septal perforator artery on CorCTA images; second, arteries with fast blood flow are considered to be more visible due to the CMR-inflow effect; third, LGE data is collected during diastole phase with emphasis on arteries. In this study, we could visualize a septal perforator artery using a slice thickness of 10 mm. First, a septal perforator artery is anatomically located along the short axis of the myocardium. Furthermore, by nullifying the myocardial signal using the IR sequences, it was possible to visualize the septal perforator artery running inside the myocardium at high signal and obtain high contrast. For these reasons, we considered that the septal perforator artery could be clearly depicted on the CMR short-axis slice, which has a lower spatial resolution than CorCTA.

It is clinically important to differentiate LHI from mid-wall fibrosis in DCM. In general, linear delayed enhancement of DCM is found “mid” of the septum myocardium (endo- and epi-cardial sparing) and extending from the anterior to the inferior wall (noncoronary territory distribution) [17]. There was excellent agreement between the pathological location of the mid-wall fibrosis and the premortem location of the mid-wall LGE [17]. On the other hand, anterior septal perforator arteries anatomically originate from the proximal part of the LAD and irrigate two-thirds of the upper part of the interventricular septum [23]. Furthermore, it has been reported that, in the short axis of cardiovascular CT, an anterior septal perforator vein runs on the endo-cardial side in the myocardium, and an anterior septal perforator artery runs on the epi-cardial side in the myocardium [21]. Based on the anatomical information, we present the LGE patterns of LHI and DCM considering the three abovementioned points. The mid-wall fibrosis is relatively specific for DCM and regarded as an important indicator of poor prognosis. Therefore, it is very important to distinguish the LHI found in the septum and mid-wall fibrosis in DCM. In DCM patients with mid-septal fibrosis, no apparent linear structure could be delineated as the normal septal perforator artery. Myocardial thinning and a range of mid-wall fibrosis may be involved in this phenomenon, but this result alone cannot clarify this in detail. As the number of comparison DCM patient was very small, further study is needed. In addition, improving the spatial resolution of delayed contrast images may allow differentiation between the septal perforator artery and mid-wall fibrosis.

The present study has several limitations. First, LGE was acquired with only IR sequences, and not with a phase-sensitive inversion-recovery (PSIR) method. PSIR techniques have the benefits of a high contrast-to-noise ratio and accurate depiction of the enhanced region [24]. Therefore, we speculate that PSIR techniques can potentially improve the detectability of the septal LHI. Second, only 1 reader examined the cases; thereafter, a second reader confirmed the assessment without an independent reading.

Finally, awareness of septum high intensity areas in LGE images is important for interpretation of imaging findings. Radiologists familiar with this finding may be able to avoid unnecessary examinations and follow-ups after CMR.

## Conclusions

The LHI signal observed in the basal anterior septum in short-axis CMR LGE images may reflect the contrast enhancement of the anterior septal perforator arteries. It is necessary to interpret this septum high intensity against knowledge of anatomic structure, to avoid misinterpretations of LGE and prevent misdiagnosis.

## Data Availability

Not applicable.

## Abbreviations

bSSFP: Balanced steady state free precession
CAD Coronary artery disease CMR: Cardiovascular magnetic resonance
CorCTA: Coronary computed tomography angiography
CT: Computed tomography
DCM: Dilated cardiomyopathy
ECG: ElectrocardiogramIR, Inversion recovery
IR Inversion recovery LGE: Late gadolinium enhancement
LHI: Linear high intensity
LV: Left ventricle/left ventricular
MIP: Maximum image projection
PSIR: Phase sensitive inversion recovery
RV: Right ventricle/right ventricular
TI: Inversion time
